# Modulation of Immune Responses by Extracellular Vesicles From Retinal Pigment Epithelium

**DOI:** 10.1167/iovs.15-18353

**Published:** 2016-08-15

**Authors:** Jared E. Knickelbein, Baoying Liu, Anush Arakelyan, Sonia Zicari, Susan Hannes, Ping Chen, Zhiyu Li, Jean-Charles Grivel, Benjamin Chaigne-Delalande, H. Nida Sen, Leonid Margolis, Robert B. Nussenblatt

**Affiliations:** 1 Laboratory of Immunology, National Eye Institute, National Institutes of Health, Bethesda, Maryland, United States; 2 Section of Intercellular Interactions, Eunice Kennedy-Shriver National Institute of Child Health and Human Development, Bethesda, Maryland, United States

**Keywords:** extracellular vesicles, retinal pigment epithelium, monocytes, T cells, immune privilege

## Abstract

**Purpose:**

Extracellular vesicles (EV), such as exosomes, are important mediators of intercellular communication and have been implicated in modulation of the immune system. We investigated if EV released from retinal pigment epithelium (RPE) modulate immune responses in vitro.

**Methods:**

Extracellular vesicles were isolated from ARPE-19 cultures stimulated or not with the inflammatory cytokines IL-1β, IFN-γ, and TNF-α. Isolated EV were characterized by nanoparticle flow and Western blot analyses. Retinal pigment epithelium–derived EV were cultured with human peripheral blood mononuclear cells, which were assayed for T-cell proliferation by ^3^H-thymidine incorporation. Retinal pigment epithelium–derived EV were also independently cultured with enriched lymphocytes or monocytes. Cell phenotype and cell death were evaluated by flow cytometric analysis. Cytokine levels were assayed in culture supernatants by multiplex bead analysis.

**Results:**

The concentration of ARPE-derived EV from cytokine-stimulated cultures was slightly higher than from nonstimulated cultures. The size of EV was approximately 100 nm in both groups. Extracellular vesicles from both nonstimulated and cytokine-stimulated ARPE-19 significantly inhibited T-cell proliferation without affecting T-cell viability. Culture of EV from nonstimulated ARPE-19 with undifferentiated human monocytes induced an immunoregulatory phenotype with a significantly higher percentage of CD14^++^CD16^+^ monocytes and upregulation of TGF-β1. Culture of EV from cytokine-stimulated ARPE-19 cells with human monocytes induced upregulation of several proinflammatory cytokines and monocyte death.

**Conclusions:**

Retinal pigment epithelium cells constitutively secrete EV in the size range of exosomes, with increased release from RPE cells stimulated with inflammatory cytokines. Extracellular vesicles from both nonstimulated and cytokine-stimulated RPE inhibited T-cell stimulation. Extracellular vesicles from nonstimulated ARPE-19 cells promoted an immunoregulatory CD14^++^CD16^+^ phenotype in human monocytes, while EV from cytokine-stimulated ARPE-19 cells caused human monocyte death. These findings suggest that RPE cells use EV to induce a downregulatory immune environment under homeostatic conditions. In an inflammatory milieu, RPE-derived EV may mitigate a potentially harmful inflammatory response through killing of monocytes.

In the healthy eye, cells of the retinal pigment epithelium (RPE) form a monolayer that abuts the photoreceptor tips apically and Bruchs' membrane basolaterally. Retinal pigment epithelium cells are critical to the normal function of overlying photoreceptors and, hence, for normal vision. Retinal pigment epithelium cell loss (e.g., geographic atrophy) results in severe vision loss and is associated with disruption of overlying outer retinal structures as well as abnormalities of the underlying choriocapillaris.^1–3^ Retinal pigment epithelium cells may become damaged during degenerative (e.g., AMD) or inflammatory (e.g., various forms of posterior uveitis) eye diseases and are incapable of regeneration under normal conditions in vivo.^4^ In the steady state, RPE cells possess immunosuppressive properties and presumably contribute to the inhibition of inflammatory damage to the retina and surrounding RPE cells. These properties include suppression of T-cell activation and induction of apoptosis in activated T cells.^5,6^ Furthermore, RPE cells have been shown to induce FasL and IL-10 production in monocytes through soluble factors.^7,8^

Recently, RPE cells have been shown to release extracellular vesicles (EV), including exosomes, considered to be important for intercellular communication.^9,10^ Exosomal markers have been identified in drusen from human eyes with AMD.^9^ A distinct pattern of exosomal proteins was observed in aqueous fluid from patients with AMD compared with age-matched controls, suggesting exosomal proteins could potentially be used as biomarkers for ocular disease.^11^

Extracellular vesicles are known to modulate immune responses.^12^ Depending on the inflammatory environment of the originating cell, EV may possess pro- or anti-inflammatory functions.^13^ The effects of RPE-derived EV on ocular immune responses remain unexplored. In the current study, we investigated if EV released from RPE cells, either in homeostatic or inflammatory environments, alter T-cell responses and monocyte phenotype in vitro.

## Materials and Methods

### Patients

Peripheral blood samples were obtained from noninfectious uveitis patients at the National Eye Institute Uveitis clinic (Bethesda, MD, USA). The study was approved by the institutional review board of the National Institutes of Health (NIH; Bethesda, MD, USA) and conformed to the tenets of the Declaration of Helsinki. Written informed consent was obtained from all patients.

### EV Isolation and Characterization

Extracellular vesicles were isolated from 10 mL of ARPE-19 culture supernatant (as below) using the ExoQuick TC isolation kit (System Biosciences, Palo Alto, CA, USA). Isolated EV were resuspended in 300 μL PBS. Extracellular vesicles size and concentration were measured with a NanoSight NS300 nanoparticle analyzer (Malvern Instruments, Malvern, UK). Additionally, isolated EV were analyzed by flow cytometry following incubation with mouse anti-human CD81 (clone: 5A6, product #: 349502; BioLegend, San Diego, CA, USA)-conjugated magnetic nanoparticles, as previously described.^14^ Briefly, EV were labeled with BODIPY-FITC and captured with anti-human CD81-coated magnetic nanoparticles (Ocean NanoTech, San Diego, CA, USA) prior to flow cytometric analysis. Megamix-Plus SSC beads (Biocytex, Marseille, France) were included for sizing, and fluorescently labeled counting beads (eBioscience, San Diego, CA, USA) were included to estimate EV concentration.

### Cells and Culture

ARPE-19 cells (CRL-2302) were purchased from ATCC (Manassas, VA, USA) and maintained according to ATCC recommendations. Peripheral blood mononuclear cells (PBMC) were isolated from the blood of uveitis patients by Ficoll gradient centrifugation. Elutriated human monocytes and lymphocytes from volunteer donors were obtained from the NIH Clinical Center Blood Bank.

ARPE-19 cells were cultured in T25 flasks in Dulbecco's modified Eagle's medium (DMEM; Gibco, Carlsbad, CA, USA) medium and 10% fetal bovine serum (FBS). At approximately 95% confluence, the cells were washed and cultured in 10 mL DMEM medium and 10% FBS with or without the inflammatory cytokines IL-1β, IFN-γ, and TNF-α (10 ng/mL each) for 48 hours. The amount of FBS was identical in all culture conditions. ARPE-19 cells were assayed by flow cytometry for viability by Annexin V/Propidium iodide (PI) staining and separately for the expression of surface and intracellular CD81 (Catalog #: 561956; BD, Franklin Lakes, NJ, USA).

The indicated volumes of RPE-derived EV (isolated as above) were cultured with PBMC (1 × 10^5^ cells/well) for 5 days, and T-cell proliferation was measured by ^3^H-thymidine incorporation. Alternatively, ARPE-19–derived EV were incubated for the indicated times with elutriated human lymphocytes or monocytes (0.5–1.0 × 10^6^ cells/well in RPMI with 10% FBS), which were then phenotypically analyzed by flow cytometry.

### Western Blot

For immunoblotting, EV samples were washed in PBS and immediately lysed in RIPA buffer (Thermo, Carlsbad, CA, USA) supplemented with phosphatase/protease inhibitor cocktail (Thermo). Protein concentrations quantitated by BCA assay (Thermo) were comparable in all the samples. Twenty micrograms of lysates were separated by SDS-PAGE and transferred on nitrocellulose (Bio-Rad, Hercules, CA, USA). The membrane was blocked with Odyssey blocking buffer (LI-COR Biosciences, Lincoln, NE, USA) for 1 hour before incubating with anti-ApoA1 (clone 5F4, Catalog #: 3350; Cell Signaling, Danvers, MA, USA), anti-ApoB11 (Catalog #: AF3620; R&D, Minneapolis, MN, USA), or anti-CD63 (Clone H5C6, Catalog #: 353013; Biolegend) antibodies overnight. Secondary Alexa Fluor 680-conjugated anti-Goat IgG (LI-COR Biosciences) and IRDye800-conjugated anti-Mouse IgG (LI-COR Biosciences) were incubated for 1 hour. Blots were imaged using an Odyssey Infrared Imaging System (LI-COR Biosciences).

### Cell Staining and Flow Cytometry

Flow cytometric analysis was performed as previously described.^15^ Monoclonal antibodies purchased from BD against human CD14 (Catalog #: 555399), CD16 (Catalog #: 555406), CD81 (Catalog #: 561956) were used. 4′,6-diamidino-2-phenylindole (DAPI) staining was used to assess viability. Samples were analyzed on a MACSQuant cytometer (Miltenyi Biotec, San Diego, CA, USA), and data were analyzed with FLOWJO software (Tree Star, Inc., Ashland, OR, USA).

### Cytokine Analysis

Cytokine levels were assayed in monocyte culture supernatants by multiplex bead analysis according to the manufacturer's instructions (Luminex kits from EMD Millipore, Billerica, MA, USA).

### Statistical Analysis

Where appropriate, ANOVA or Student's *t*-test were used to calculate differences between groups. *P* less than 0.05 was considered statistically significant.

## Results

### Characterization of RPE-Derived EV

**Figure 1:**
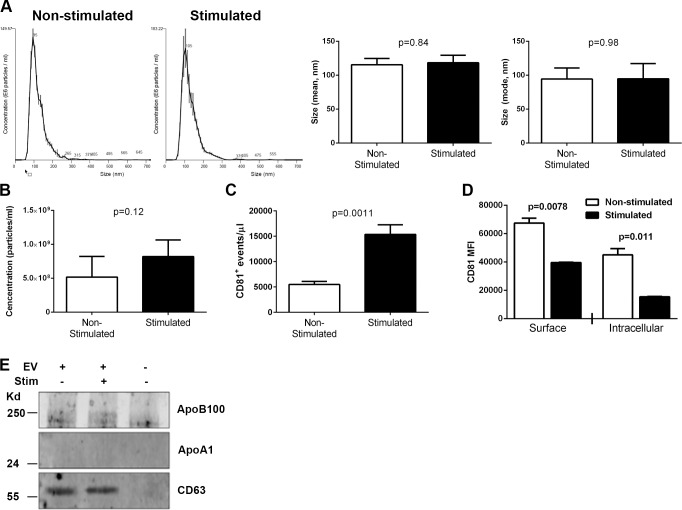
Characterization of EV released by RPE cells stimulated or not with inflammatory cytokines. Concentration (**A**) and size (**B**) of EV isolated with ExoQuick from ARPE-19 cells stimulated or not with 10 ng/mL each IL-1β, IFN-γ, and TNF-α for 48 hours as measured by NanoSight nanoparticle analysis. Presented are the mean ± SEM from three independent experiments (*n* = 5 per group). Student's *t*-test did not reveal significant differences between groups for concentration (*P* = 0.12) or size (*P* = 0.84 for mean, *P* = 0.98 for mode). (**C**) Extracellular vesicles isolated from ARPE-19 with ExoQuick were labeled with BODIPY-FITC and captured with anti-human CD81-coated magnetic nanoparticles prior to flow cytometric analysis. Counting beads were included to calculate EV concentration. Presented are the mean ± SEM from three independent experiments (*n* = 5 per group). (**D**) ARPE-19 cells stimulated or not with 10 ng/mL each IL-1β, IFN-γ, and TNF-α for 48 hours were stained for surface and intracellular CD81 prior to flow cytometric analysis (*n* = 2 per group). Data representative of three independent experiments. (**E**) Extracellular vesicles isolated from ARPE-19 with ExoQuick were digested and analyzed for the apolipoprotein markers ApoB100 and ApoA1, as well as the extracellular vesicle marker CD63 by Western blot.

Extracellular vesicles were isolated from culture supernatants of ARPE-19 cells stimulated or not with IL-1β, IFN-γ, and TNF-α. Regardless of cytokine stimulation, greater than 95% of ARPE-19 cells were viable as evaluated by Annexin V/PI staining (data not shown). The average size of EV evaluated by the NanoSight assay was approximately 100 nm and did not differ between the groups (*P* = 0.84 for mean, *P* = 0.98 for mode; Fig. 1A). NanoSight analysis also revealed slightly more EV in cultures of ARPE-19 cells stimulated with inflammatory cytokines compared with nonstimulated ARPE-19 cells, but this difference was not statistically significant (*P* = 0.12; Fig. 1B). To confirm that human ARPE-19-derived EV were present in our EV preparations, as opposed to those from FBS, we incubated the EV preparations with magnetic nanoparticles coated with anti-human CD81, a tetraspanin protein enriched in exosomes, prior to flow cytometric analysis (Fig. 1C). These data revealed significant populations of CD81^+^ EV of appropriate sizes from both cytokine-stimulated and nonstimulated ARPE-19 cultures. Consistent with the NanoSight concentration analysis, more EV were observed in supernatants from cytokine-stimulated compared with nonstimulated ARPE cells. Therefore, EV from the FBS were likely contributing to the NanoSight analysis. To further support altered EV metabolism in RPE stimulated with inflammatory cytokines, we assessed the level of surface and intracellular CD81 on and within ARPE-19 cells stimulated or not with IL-1β, IFN-γ, and TNF-α. Stimulation of ARPE-19 cells with these inflammatory cytokines resulted in significantly reduced expression of surface and intracellular CD81 compared with nonstimulated controls, suggesting increased release of EV from ARPE-19 cells stimulated with IL-1β, IFN-γ, and TNF-α with resultant shedding of CD81 (Fig. 1D). To determine if the preparations of RPE-derived EV also contained lipoprotein particles, which can be coisolated during ExoQuick precipitation and may also modulate immune responses,^16,17^ Western blot analysis was performed for the lipoprotein markers ApoB100 and ApoA1 as well as the EV marker CD63. As seen in Figure 1E, little if any ApoB100 and ApoA1 were detected in the EV preparations, while CD63 was strongly present.

### RPE-Derived EV Inhibit T-Cell Proliferation

**Figure 2:**
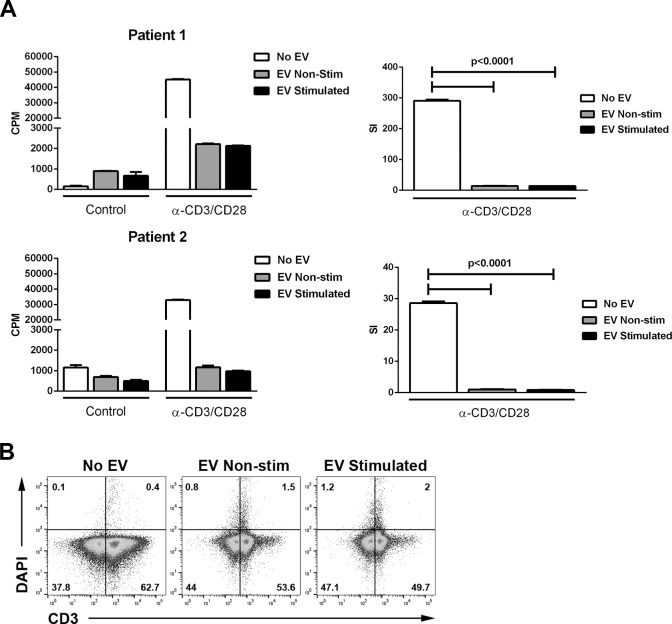
Retinal pigment epithelium–derived EV inhibit T-cell proliferation but do not induce T-cell death. (**A**) Extracellular vesicles were isolated from ARPE-19 cells stimulated (EV- stimulated; 5.7e^8^ ± 1.1e^8^ particles/mL) or not (EV: nonstimulated; 3.0e^8^ ± 5.4e^7^ particles/mL) with IL-1β, IFN-γ, and TNF-α (10 ng/mL of each), and 5 to 50 μL (data from 50 μL are shown) of EV isolate were cultured with PBMC isolated from peripheral blood of uveitis patients (100,000 cells/well in RPMI with 10% FBS). T-cell stimulation was measured as described in the Methods section. Stimulation index (SI) was calculated by dividing the counts per minute (CPM) of experimental groups by that of the control nonstimulated group. *P* values calculated by ANOVA with Tukey's multiple comparisons test. (**B**) Elutriated human lymphocytes (0.5–1 × 10^6^ cells/well) were cultured alone or with 75 μL EV released by resting (1.2e^9^ ± 6.1e^7^ particles/mL) or cytokine-stimulated (1.2e^9^ ± 3.7e^7^ particles/mL) ARPE-19 cells. At 1, 2, 3, and 4 days of culture, cells were harvested and stained with CD3 and DAPI and analyzed with flow cytometry. Data from 4-day cultures are shown. All other time points showed similar low levels of T-cell DAPI inclusion.

To investigate the effect of RPE-derived EV on T-cell responses, PBMC from patients with noninfectious uveitis were stimulated with anti-CD3 and anti-CD28 antibodies in the presence or absence of EV from resting or cytokine-stimulated ARPE-19. Extracellular vesicles from both resting and cytokine-stimulated ARPE-19 significantly reduced T-cell proliferative responses, as evaluated by ^3^H-thymidine incorporation, compared with cultures that did not receive EV (*P* < 0.0001; Fig. 2A). Reduced ^3^H-thymidine incorporation could be caused by either inhibition of T-cell proliferation or by T-cell death. To distinguish these possibilities, T cells from enriched lymphocytes cultured with EV were stained with DAPI and analyzed by flow cytometry. Neither EV from resting nor cytokine-stimulated ARPE-19 induced significant T-cell death over a culture period of 4 days (Fig. 2B).

### RPE-Derived EV Modulate Human Monocyte Phenotype and Survival

**Figure 3:**
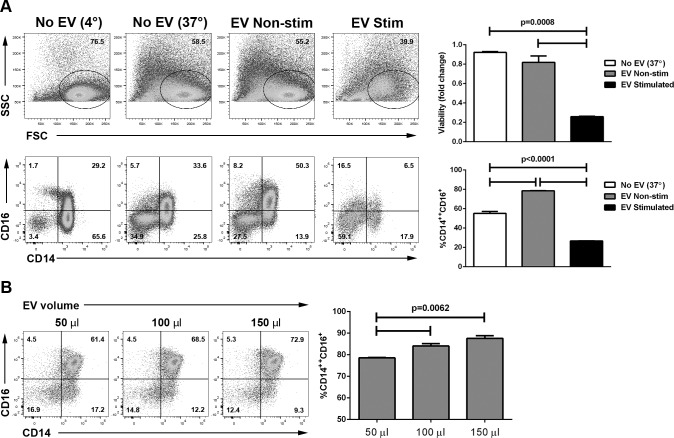
Modulation of human monocyte phenotype by ARPE-19–derived EV. (**A**) Extracellular vesicles were isolated from ARPE-19 stimulated (EV: stimulated) or not (EV: nonstimulated) with IL-1β, IFN-γ, and TNF-α 10 ng/mL each for 48 hours, and 75 μL of EV isolate (1.1e^9^ ± 4.8e^7^ particles/mL for cytokine-stimulated ARPE-19 and 9.0e^8^ ± 1.6e^7^ particles/mL for nonstimulated ARPE-19) were cultured with elutriated human monocytes (0.5–1 × 10^6^ cells/well) for 24 hours. Cells were then stained with DAPI to assess viability and for CD14 and CD16 and analyzed by flow cytometry. *Top row*: dot plots showing forward (FSC) and side scatter (SSC); graph on the *right* shows the proportion of viable cells compared with those kept at 4° during the culture period. *Bottom row*: dot plots showing CD14 and CD16 expression; graph on the *right* shows the percentage of intermediate (CD14^++^CD16^+^) monocytes of all CD14^+^ cells in the different groups. Data representative of three independent experiments. (**B**) Extracellular vesicles were isolated from ARPE-19 not exposed to cytokines, and the indicated volume of EV isolate (2.2e^8^ ± 4.0e^7^ particles/mL) was cultured with elutriated human monocytes for 24 hours. Cells were then stained for CD14 and CD16 and analyzed by flow cytometry. * *P* < 0.05; *P* values calculated by ANOVA with Tukey's multiple comparisons test.

We next explored the effect of EV on monocyte phenotype and survival. Human monocytes may be classified based on their surface expression of CD14 and CD16 into three groups: classical (CD14^++^CD16^−^), intermediate (CD14^++^ CD16^+^), and nonclassical (CD14^+^CD16^++^).^18^ In cultures of elutriated human monocytes incubated with EV from nonstimulated ARPE-19 cells, no significant monocyte death was observed (Fig. 3A). The fraction of CD14^++^CD16^+^ cells was significantly larger than in control monocyte cultures that did not receive EV (*P* < 0.05; Fig. 3A), and the enrichment of CD14^++^CD16^+^ cells directly correlated with the number of EV added to the culture (Fig. 3B). Extracellular vesicles from cytokine-stimulated ARPE-19 cells induced significant monocyte death (∼75%; *P* < 0.05; Fig. 3A), which directly correlated with the number of EV added to the culture over a 3-fold dilution (data not shown). The remaining monocytes were mostly of the classical phenotype (Fig. 3A).

### RPE-Derived EV Modulate Cytokine Release in Human Monocyte Cultures

**Figure 4:**
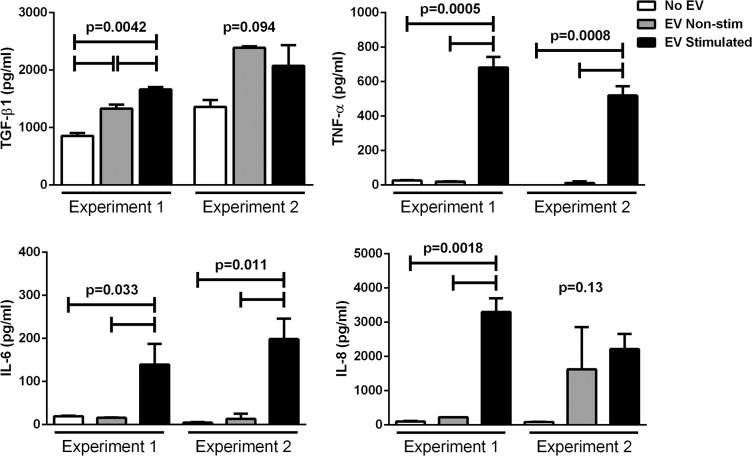
Cytokine levels in cultures of human monocytes with ARPE-19–derived EV. Extracellular vesicles isolated from ARPE-19–stimulated (EV: stimulated) or not (EV: nonstimulated) with IL-1β, IFN-γ, and TNF-α were cultured with elutriated human monocytes for 24 hours as in Figure 3, and supernatants were analyzed for the indicated cytokines by multiplex bead analysis. Results from two independent experiments are shown. *P* values calculated by ANOVA with Tukey's multiple comparisons test or Student's *t*-test.

The concentration of TGF-β1, which has several immunosuppressive functions, in supernatants from monocyte cultures treated with EV released by nonstimulated APRE-19 cells was higher than in supernatants from untreated cells (Fig. 4). The concentrations of the inflammatory cytokines TNF-α, IL-6, and IL-8 in supernatants from monocyte cultures treated with EV released by nonstimulated APRE-19 cells and in supernatants from untreated cells were similarly low. In contrast, supernatants from monocytes cultured with EV released by cytokine-stimulated ARPE-19 contained significantly higher levels of proinflammatory cytokines compared with supernatant from both untreated monocytes and monocytes cultured with EV released by nonstimulated ARPE-19 (Fig. 4).

## Discussion

In the steady state, RPE cells possesses several immunosuppressive properties.^7,8,19^ However, the mechanisms by which RPE cells communicate with adjacent cells to mediate these immunosuppressive effects are not fully understood. Based on the data presented herein, we suggest that EV may be a mediator of these immunosuppressive functions by demonstrating that RPE-derived EV differentially modulate T-cell responses and monocyte phenotype depending on the inflammatory milieu of the RPE cells.

It is now well-established that EV, in particular exosomes originating from the endosomal pathway and typically of approximately 100 nm in dimeter,^12^ are important mediators of intracellular communication.^20^ Several studies have now demonstrated the release of exosomes from RPE cells.^9,10,21^ Here, we compared the size, concentration, and immune-modulatory function of EV released by RPE stimulated with the inflammatory cytokines IL-1β, IFN-γ, and TNF-α with those released by nonstimulated RPE. Extracellular vesicles released from RPE-stimulated or not with inflammatory cytokines are in the size range of exosomes. In examining the concentration of EV released from RPE cells, bulk analysis with a nanoparticle analyzer, which measures particles derived from both RPE and FBS, revealed similar concentrations of EV from stimulated and nonstimulated ARPE-19 cells. However, analysis by flow cytometry using human-specific antibodies revealed an approximately 3-fold increase in the concentration of EV released from cytokine-stimulated ARPE-19 compared with nonstimulated controls. Furthermore, flow cytometric analysis demonstrating reduced levels of CD81, an EV marker, on and within ARPE-19 stimulated with inflammatory cytokines compared with nonstimulated controls suggests that stimulated ARPE-19 cells are secreting more EV. These results are consistent with previous reports demonstrating increased exosome release from RPE cells exposed to oxidative stress.^22^

Lipoprotein particles may be coisolated with EV during ExoQuick precipitation and may also modulate immune responses.^16,17^ Therefore, we performed Western blot analysis on the EV preparations from both cytokine-stimulated and nonstimulated ARPE-19 cells for the lipoprotein markers ApoB100 and ApoA1 as well as the EV marker CD63. Little if any ApoB100 and ApoA1 were detected in the EV preparations, while CD63 was strongly present. Therefore, we conclude that lipoprotein particle coisolation with EV from ARPE-19 cultures is unlikely contributing significantly to the immunomodulatory effects we have demonstrated.

We evaluated the effects of RPE-derived EV on T cells and monocytes, two cell types important for intraocular immune responses. Extracellular vesicles from both resting and cytokine-stimulated RPE inhibited the proliferation of T cells in PBMC cultures. Importantly, RPE-derived EV did not induce T-cell death. We next tested the ability of RPE-derived EV to alter the phenotype and activation status of normal human monocytes. Human monocytes exposed to EV released by nonstimulated RPE cells resulted in an enrichment of the CD14^++^CD16^+^ intermediate monocyte population without affecting monocyte viability. CD14^++^CD16^+^ intermediate monocytes from noninfectious uveitis patients, unlike classical and nonclassical monocytes, have strong immunoregulatory effects, such as a reduced capacity to activate T-cell effector functions, as we showed earlier.^15^ Furthermore, the immunosuppressive cytokine TGF-β1 was upregulated in supernatants from monocyte cultures with EV released by nonstimulated RPE but not in control cultures that did not contain EV. These findings suggest that resting ARPE-19 cells, which are known to possess immunosuppressive properties, may use EV to mediate these functions. This may represent one mechanism by which RPE maintains a homeostatic immunosuppressive environment to protect neighboring RPE as well as adjacent photoreceptors from inflammatory damage. Moreover, EV from other cell types, such as immunosuppressive dendritic cells, have been shown to downregulate immune responses.^23,24^

Incubation of human monocytes with EV released by cytokine-stimulated ARPE-19 cells lead to monocyte cell death and upregulation of several proinflammatory cytokines. Exosomes released from ARPE-19 exposed to oxidative stressors were previously reported to preferentially upregulate proteins associated with apoptosis, such as Bak and Smac/Diablo, and downregulate proteins associated with cell proliferation and survival, such as Akt and Src family proteins.^22^ We hypothesize that the EV released by RPE cells exposed to inflammatory conditions are modified to express proapoptotic molecules that may reduce the number of infiltrating immune cells in an attempt to abrogate immune-mediated damage to surrounding RPE cells and photoreceptors. The upregulation of inflammatory cytokines may have been a protective response from the monocytes in an attempt to prevent apoptosis, because inflammatory cytokines, including TNF-α, have been shown to reduce monocyte apoptosis.^25^ Furthermore, the differential effect of EV from nonstimulated and cytokine-stimulated RPE cells on monocytes may explain the suppression of T-cell responses by both types of EV. Extracellular vesicles from nonstimulated RPE cells may suppress T-cell stimulation through enrichment of immune regulatory CD14^++^CD16^+^ monocytes, while EV from cytokine-stimulated RPE cells may inhibit T-cell stimulation due to a lack of APC as a result of monocyte death.

Taken together, our results indicate that RPE release EV that suppress immune responses under both resting and inflammatory conditions. Under resting conditions, RPE constitutively release EV that inhibit T-cell proliferation and render monocytes immunosuppressive without affecting their survival. Under inflammatory conditions, RPE release EV that inhibit T-cell proliferation without affecting T-cell viability but that induce monocyte cell death, perhaps to dampen an actively destructive immune response. These results suggest that EV secretion may be an important mechanism used by RPE cells to exert their known immunomodulatory effects.
